# New Insights from Elucidating the Role of LMP1 in Nasopharyngeal Carcinoma

**DOI:** 10.3390/cancers10040086

**Published:** 2018-03-21

**Authors:** Kathy H. Y. Shair, Akhil Reddy, Vaughn S. Cooper

**Affiliations:** 1 Cancer Virology Program, University of Pittsburgh Medical Center, Pittsburgh, PA 15213, USA; ANR156@pitt.edu; 2 Department of Microbiology and Molecular Genetics, and Center for Evolutionary Biology and Medicine, University of Pittsburgh, Pittsburgh, PA 15219, USA; vaughn.cooper@pitt.edu

**Keywords:** Epstein-Barr virus, LMP1, epithelial infection, nasopharyngeal carcinoma

## Abstract

Latent membrane protein 1 (LMP1) is an Epstein-Barr virus (EBV) oncogenic protein that has no intrinsic enzymatic activity or sequence homology to cellular or viral proteins. The oncogenic potential of LMP1 has been ascribed to pleiotropic signaling properties initiated through protein-protein interactions in cytosolic membrane compartments, but the effects of LMP1 extend to nuclear and extracellular processes. Although LMP1 is one of the latent genes required for EBV-immortalization of B cells, the biology of LMP1 in the pathogenesis of the epithelial cancer nasopharyngeal carcinoma (NPC) is more complex. NPC is prevalent in specific regions of the world with high incidence in southeast China. The epidemiology and time interval from seroconversion to NPC onset in adults would suggest the involvement of multiple risk factors that complement the establishment of a latent and persistent EBV infection. The contribution of LMP1 to EBV pathogenesis in polarized epithelia has only recently begun to be elucidated. Furthermore, the LMP1 gene has emerged as one of the most divergent sequences in the EBV genome. This review will discuss the significance of recent advances in NPC research from elucidating LMP1 function in epithelial cells and lessons that could be learned from mining LMP1 sequence diversity.

## 1. Introduction

In 1964, Tony Epstein and Yvonne Barr observed herpesvirus-like particles in electron micrographs of Burkitt lymphoma cells [1]. This was the founding discovery that eventually associated a ubiquitous-herpesvirus (Epstein-Barr virus, EBV) with human cancers [2,3,4]. A series of landmark serology and molecular virology studies in the late 1960s–1970s further established that latent EBV infection is intimately linked to some epithelial carcinomas and B-cell lymphomas in both immunocompetent and immunosuppressed hosts, and was not simply a bystander infection [2,5,6,7]. The immortalization of primary B-lymphocytes by EBV infection generating lymphoblastoid cell lines (LCLs) is the in vitro assay for assessing EBV immortalizing properties [8]. Recombinant EBV bacterial artificial chromosome (BAC) technology combined with the EBV-immortalization assay has enabled the testing of latent transcripts (coding and non-coding) for the immortalization and continued viability of LCLs [9,10]. Latent membrane protein 1 (LMP1), expressed in type II and III latency programs, is one of at least six latent transcripts required for EBV-mediated B-cell immortalization [8,11,12]. In non-lymphoid cells, LMP1 is also considered a major EBV oncoprotein that promotes transforming properties in Rat-1 fibroblasts and epithelial cell lines, conferring the loss of contact inhibition, anchorage-independent cell growth, increased motility and tumors in nude mice [13,14,15]. 

Despite the strong association of LMP1 expression to the immortalizing and pro-survival properties in B-lymphocytes, the molecular basis to explain how latent EBV infection can be a co-factor in the EBV-associated epithelial cancer nasopharyngeal carcinoma (NPC) has been more enigmatic [6,16]. While experiments in epithelial cells demonstrate that LMP1 expression has oncogenic potential, NPC cells express variable levels of LMP1 protein with foci of positivity within the NPC tumor [17,18]. However, LMP1 transcript is consistently detected across NPC tumors which would suggest that low levels of LMP1 may be sufficient to confer oncogenic properties [6,17]. The lower LMP1 expression levels in NPCs may be more comparable to the non-cytotoxic levels expressed in retroviral- and lentiviral-transduced stable cell lines with LTR-driven expression systems [19,20]. Arguably one of the most important observations in associating EBV infection to NPC is that EBV infection is clonal in early preinvasive lesions and in the NPC tumor [21,22]. These conclusions on EBV clonality are based on a defining genomic structure created by the fusion of tandem terminal repeats, which would be consistent with the hypothesis that EBV infection is present at the inception of neoplastic transformation [21]. However, EBV infection in differentiated stratified epithelia is primarily lytic with production of progeny virus [23]. Therefore, several important questions remain in elucidating how EBV maintains latency and the origin of clonal EBV genomes in the NPC tumor [7,24,25]. Despite knowledge of LMP1 oncogenic signaling properties, the biological function of LMP1 in EBV pathogenesis has yet to be fully elucidated [15,17,26,27].

LMP1 is a six-pass transmembrane protein that is primarily characterized for its effects on cellular signaling pathways via protein-protein interactions in plasma and cytosolic membranes but is not known to possess intrinsic enzymatic activity [28]. The pleiotropic signaling properties of LMP1 have been extensively reviewed [6,14,15,17,28]. This review will focus on the latest developments on EBV epithelial infection models, discuss the implications of LMP1 sequence diversity, and summarize LMP1 effects on nuclear and extracellular processes.

## 2. Epithelial Infection Models

EBV infection is life-long and during chronic infection, EBV resides in memory B-lymphocytes expressing a limited number of latent transcripts with sporadic reactivation and transmission from saliva [29]. Although EBV latent and lytic infections are associated with epithelial diseases including the latency-associated cancers NPC and gastric carcinoma, and the permissive disease oral hairyleukoplakia (OHL) in acquired immunodeficiency syndrome (AIDS) patients, the question of whether EBV is also maintained as an epithelial reservoir is debated [24,30,31]. Paradoxically, in asymptomatic seropositive individuals, it has been difficult to isolate infected (latent or lytic) epithelial cells from the oral epithelium [22,32,33,34]. This has been ascribed to the likely rare occurrence of infected cells which may exist as foci of infected cells [35]. NPC tumors express a type II latency program including EBNA1, LMP1, LMP2A, LMP2B, EBER1/2 and the BART transcript that encode the abundantly transcribed viral microRNAs (miRNAs [6,36]). Epithelial infection models using the prototypic B95-8 reference strain or M81 epithelial-tropic strain, or the Burkitt-lymphoma derived recombinant Akata strain EBV, consistently results in an acute lytic and productive infection [23,37,38,39,40]. Following an acute period of permissive infection, latently-infected outgrowing cell clones can be selected and cultured [41,42,43]. Tissue specific differences are also notable. In comparison to the latent infection in NPC tumors, the AIDS-associated permissive infection OHL develop in the tongue and oral epithelium, but these remain as lytic lesions and do not develop into carcinomas [44,45]. The return of OHL lesions after eradication of EBV permissive replication by valacyclovir treatment would support the possible existence of a persistent (perhaps latent or uncleared) oral reservoir [46,47,48]. Although few studies have documented EBV infection in nasal and airway epithelial cells, likely due to the limited sampling material from the nasopharynx compared to the more accessible oral epithelial tissue, EBV infection is almost always latent and associated with NPC tumors [6,24,33]. These contrasting clinical observations of a permissive infection in oral epithelia compared to the latent infection in NPC and gastric carcinoma would suggest that tissue-specific differences should be considered when interpreting infection outcomes in epithelial infection models.

Compared to the efficient infection in B cells, de novo EBV infection in epithelial cells is relatively inefficient [24]. Before the discovery of an epithelial-tropic virus strain M81, modifications to the infection method by co-culture with producer B cells or, transfer infection by capturing virions on the surface of primary B cells or, cell-free virus concentration, would enhance EBV infection in immortalized and primary epithelial cells to some degree [37,49,50,51]. Experimental variables including the addition of EGF and TGF**β**, expression of nonmuscle myosin heavy chain IIA and neuropilin 1, and fusion of the gHgL 2-part complex with the cellular integrins αVβ6 and αVβ8 have all been proposed to be important criteria for epithelial infection in vitro [42,52,53,54]. The details of EBV entry and fusion have been reviewed elsewhere [55,56]. Perhaps the most important advancement that has led to the recent discovery of the EBV receptor, Ephrin A2, in epithelial cells is that pre-treatment of immortalized nasopharyngeal epithelial cells with EGF enhances EBV infection with cell-free virus (at a multiplicity of infection of 1000) by more than 2-fold, reaching up to 45% infection efficiency [57]. At the same time, an independent study also uncovered Ephrin A2 as the primary EBV receptor for epithelial cells by deductive reasoning [58]. The authors reasoned that HEK293 and AGS gastric carcinoma cells that are highly susceptible to EBV infection, would express a differentially abundant membrane-associated EBV receptor that would be absent from B cells. Together, these molecular discoveries and methodologies have overcome considerable unknowns that are anticipated to accelerate EBV research in the context of understanding viral persistence and pathogenesis in epithelial cells, and perhaps in the early events leading to EBV-associated epithelial cancers.

**Figure 1:**
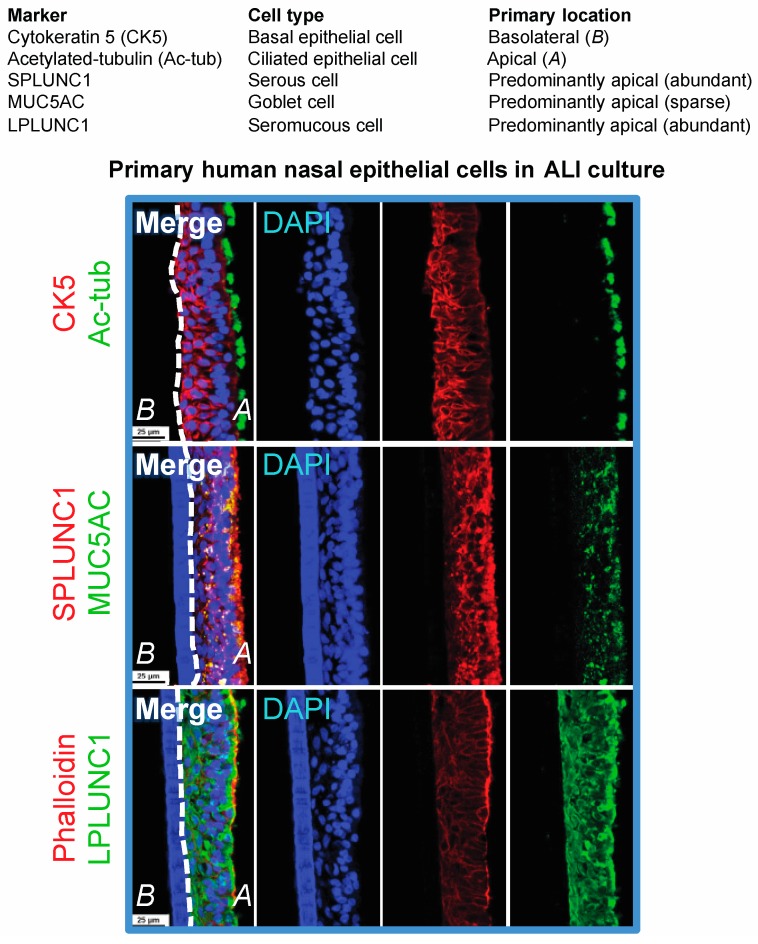
Confocal images of primary human nasal epithelial air-liquid interface (ALI) culture cryosections immunostained for cellular markers. Images were acquired on a Nikon A1 point scanning confocal microscope, processed with NIS Elements, and Z-stack 3D renderings are projected in the x-y plane.

While there have been significant improvements in facilitating the infection of immortalized and primary epithelial cells, advancements in developing epithelial cell culture models that produce infectious progeny virus and are amenable to molecular virology (DNA, RNA, protein, and virus tittering) analysis techniques are also necessary. Apart for HEK293 cells that produce high titer infectious virus, conventional chemical induction methods by 12-*O*-tetradecanoylphorbol 13-acetate (TPA) and sodium butyrate treatment (or other histone deacetylase [HDAC] inhibitors such as SAHA), or by transfection of the EBV immediate-early switch protein “Z” and the late glycoprotein “gB”, do not always result in efficient production of progeny virus that spread despite induction of lytic genes [9,30,59]. Cell lines (primary or immortalized) can be subjected to a variety of molecular virology techniques including immunoblotting, RNA-seq, Southern blotting to differentiate latent fused episomal genomes from linear replicating genomes, titering by quantitative polymerase chain reaction (qPCR) of encapsidated DNase-resistant EBV genomes, and scoring for infectious units by the green Raji assay [26,60]. Immunostaining methods that afford single cell resolution are also crucial when studying diversified cell types representative of polarized primary epithelial tissue, or when assessing differences in infection outcome from the basolateral and apical surfaces [23]. Single cell analysis techniques are particularly relevant for the study of NPC pre-neoplastic mechanisms that could result from infrequent clonal events that may be cell-type specific. The nasal airway epithelium consists of diversified cell types including basal, ciliated, serous, goblet, and seromucous cells (Figure 1). 

A few polarized culture models have proven invaluable and are amenable to DNA, RNA and protein analyses but could also be analyzed with single cell resolution by immunostaining and in-situ hybridization methods [23,39,61]. The organotypic raft culture method adapted from virus replication studies in HPV, enriches for and differentiates (primary or immortalized) stratified keratinocytes [62]. While the hTERT-immortalized keratinocyte organotypic raft culture model may be more readily inherited by laboratories due to cell line availability, the primary oral keratinocyte organotypic raft culture model is unique in that it results in widespread infection, and is thus also a model for virus dissemination and spread [23,39]. Furthermore, primary epithelial cells polarized on transwell membranes have demonstrated that cell-associated and cell-free virus can enter stratified epithelium via basolateral or apical surfaces [40,61]. These polarized culture techniques, whether seeding on organotypic raft inserts or transwell membranes, are based on lifting an epithelial monolayer to the air-liquid interface (ALI) in order to trigger polarization and/or stratification. The ALI polarization method has historically been used to polarize primary bronchial and nasal epithelial cells for the study of airway pathogens including viruses and biofilms [63,64,65]. The ALI culture method that preserves the diversity of cell types in primary airway epithelial cells may help to answer new questions regarding the expression and distribution (basolateral or apical) of Ephrin A2 in different donors, and potentially identify differences in EBV-infection susceptibility and outcome. While there is no clear explanation for the lack of LMP1 detection in pre-neoplastic nasopharyngeal tissue but consistent detection in preinvasive lesions (dysplastic and carcinoma in situ), culture systems that retain the diversity of cell types representative of primary tissue may help to stratify the expression of LMP1 in different cell types upon de novo infection and possibly during the early events of oncogenesis [22,26,27,66,67].

## 3. Elucidating the Role of EBV Infection and LMP1 Expression in NPC Pathogenesis

With respect to oncogenesis, infection in epithelial cells contrasts with several aspects of EBV infection in B-lymphocytes. Most notably, EBV infection is permissive in epithelial cells and therefore de novo EBV infection per se is not directly immortalizing [24]. Monoclonal EBV infection in NPC tumors would argue that EBV is present at the inception of the tumor cell; however, the long period from the time of seroconversion to the onset of NPC tumor would support that additional key cellular event(s) such as genetic mutations are likely co-factors [22,68]. There is evidence to support that there are mutational hotspots including the loss of p16 that would encourage the immortalization of primary epithelial cells to complement EBV infection [67,68]. However, a limited number of exome and genome sequencing studies have not identified unifying mutations, even in microdissected NPC tumors [69,70,71]. This suggests that the genetic composition supporting EBV oncogenesis in NPC may be variable, although the affected pathways such as regulators of NFkB signaling may be common [70,72]. Over-expression of Cyclin D1, telomerase, and Bmi-1 belonging to the polycomb family of proteins, encourages the immortalization of nasopharyngeal epithelial cells, which upon infection in vitro can be selected for latent outgrowths [66,67,73]. Paradoxically, in the absence of recombinant selection EBV infection is often evicted from de novo-infected cell lines or from explanted cultures of spontaneously immortalized NPC tumor cells infected in situ [6,24,74]. Due to these inconsistencies, careful assignment for the role of EBV infection in the pathogenesis of NPC is necessary. One important distinction of latent EBV infection in all EBV-associated cancers, compared to the integrated genomes and consequential unlicensed expression of viral oncogenes associated with other human tumor viruses such as human papillomavirus (HPV), hepatitis B virus (HBV) and Merkel cell polyomavirus (MCV), is that EBV genomes are maintained in their natural infectious configuration as extrachromosomal episomes in EBV-associated cancers [75]. This strongly argues that expression of latent transcripts including the heterogenous levels of LMP1 is highly regulated, and that pre-disposition to EBV-mediated oncogenesis cannot simply be explained by unlicensed expression of viral oncogenes or non-coding transcripts.

Compared to the consistently high-levels of LMP1 expression in B-cells, the level of LMP1 is diminished and varies greatly ranging from negligible to reliably detectable levels in epithelial infections [19,70,76]. Inducible LMP1 expression can yield a spectrum of phenotypes including acute cytotoxicity that contrast with the known oncogenic properties of LMP1 [77]. Conversely, experiments stably expressing lower levels of LMP1 in epithelial cells and Rat-1 fibroblasts is sufficient to promote oncogenic properties [20,78]. While induced cytotoxicity may illustrate the consequence of unlicensed LMP1 expression from recombinant promoters, NPC tumors typically maintain an overall low level of LMP1 expression as determined by immunohistochemistry staining [18,79]. It is likely that LMP1 regulation in epithelial infections is tightly regulated and distinct from the regulation in B cells. Indeed, in addition to the B-cell active LMP1 promoter (ED-L1), a second LMP1 TATA-less promoter located in the terminal repeats (L1-TR) producing a 3.5kb transcript that is differentially regulated by STAT3, is active in NPC tumors [80]. Much of the research on LMP1 has been dedicated to defining oncogenic signaling properties and elucidating immune recognition that is suspected to govern LMP1 sequence diversity [81]. However, recent data has begun to unravel the role of LMP1 in regulating the life cycle of EBV by facilitating lytic reactivation in differentiating epithelia [26,27]. Therefore, the subdued and highly regulated levels of LMP1 in NPC tumors are likely reflective of co-evolution with the host cell that is likely selected for at least three criteria including oncogenic, immune evasion and EBV pathogenesis properties.

## 4. LMP1 Sequence Diversity and Potential Selection

### 4.1. EBV Compartmentalization by LMP1 Strain Variants

Early studies on LMP1 sequence diversity identified conserved signaling domains termed the C-terminal activation regions (CTAR) 1, 2, and 3 that were critical for mediating LMP1-induced oncogenic signaling [82,83]. While the conserved motifs in CTARs 1, 2 or 3 have been extensively characterized for signaling and oncogenic properties, relatively few studies have tested for the consequence of LMP1 sequence diversity [17,20]. Before the advent of high-throughput sequencing (HTS), LMP1 sequence diversity was originally characterized by restriction enzyme polymorphisms or by Sanger sequencing of PCR-amplified and cloned LMP1 products [82,83,84]. One polymorphism results in the loss of a XhoI restriction enzyme site located in exon 1 of the LMP1 gene, which is also a feature of the predominant EBV strain found in endemic NPCs in Southeast China [85]. Multiple variants of LMP1 were further defined by three features in the C-terminus including a 30bp deletion, a variable number of 33bp repeats, and a 15bp insertion in one of the 33bp repeat regions in the coding sequence [82]. These LMP1 features did not however co-segregate with EBV type I or type II, which are distinguished by the sequence of the EBV nuclear antigens (EBNA2 and EBNA3) [82,86]. 

A survey of LMP1 sequence diversity identified that the C-terminus can phylogenetically distinguish at least seven LMP1 variants denoted as LMP1 strains (B95-8, China1, China2, Med+, Med−, Alaskan, North Carolina) [84]. The heteroduplex tracking assay (HTA) was developed to screen circulating LMP1 strains in oral wash and peripheral blood, as compared to the strains found in EBV-associated tumors and diseases [45,81,87,88]. The HTA assay is based on the hybridization of unique sequences in exon 3 of LMP1 to a PCR-amplified reference probe that can also measure relative strain abundance able to detect 1% of LMP1 strain variants from a mixed population [87]. While multiple LMP1 strains can be detected in throat washings and peripheral blood lymphocytes (PBLs), only one LMP1 strain is typically found in any one NPC tumor, as defined by the variable terminal repeats that differ between EBV clones [21,22,81,84]. This observation is consistent with clonal EBV infection in NPC [21,22,81,84]. China1 is the predominant LMP1 strain found in both endemic and non-endemic NPC tumors, with a detection frequency in up to 80% of endemic NPCs [81]. In most cases, the strain variants detected in the NPC tumor could also be detected at low abundance in PBLs, but the predominant LMP1 strain circulating in PBLs is frequently not the strain in the matching NPC tumor [81]. These findings from LMP1 strain variant studies in NPC and in other EBV-associated diseases are consistent with a model in which EBV infection is dynamically transmitted between the oropharynx and peripheral sites, and that specific isolates can also be compartmentalized within local and peripheral sites at least for the selection of LMP1 strains [81,87]. Furthermore, asymptomatic carriers also show compartmentalization of LMP1 strain variants with distinct sequences that could be tracked as persistent isolates in the oral cavity [88]. Collectively, LMP1 compartmentalization data help to profile intra-host EBV transmission patterns and propose that epithelial cells may indeed be an important reservoir for maintaining oral persistence [88]. More recently, HTS has consistently confirmed these original findings of LMP1 compartmentalization, but comparison to the rest of the EBV genome has further revealed that LMP1 is one of the most diversified sequences in the EBV genome which encode for more than 70 open reading frames [89,90,91]. In addition to the seven C-terminal signature amino acid residues that define the seven LMP1 strains, polymorphisms distributed in the N-terminal transmembrane domains and the C-terminal signaling domains have been noted [84]. It remains to be determined whether the growing number of deposited EBV sequences may eventually lead to the identification of tumor-specific LMP1 polymorphisms and greater confidence in elucidating the molecular basis of LMP1 evolution and selection in NPC tumors.

### 4.2. Comparison of LMP1 Sequence Derived from HTS of EBV Genomes

Target enrichment methods have been developed for HTS using PCR-amplified DNA baits or overlapping RNA baits to capture the EBV genome [90,91,92,93]. In theory, target enrichment methods can be susceptible to sequence bias from the bait library. An alternative method based on a modified Hirt extraction protocol to isolate extrachromosomal EBV genomes from infected cells, has been successfully applied to the sequencing of replicating EBV genomes from reactivated Akata and Mutu Burkitt lymphoma cell lines [94]. However, biochemical purification of EBV genomes by such methods has not been reported to be amenable for the sequencing of latent EBV genomes from infected cells or from clinical biopsies. To date, 33 whole EBV genomes derived from NPC tumors have been sequenced and are summarized in Table 1. The EBV genomes listed in Table 1 were derived from NPC patients in the form of an NPC biopsy (GD2, M81, D3201.2, HKNPC1-9, HN1-18), an NPC cell line (C666-1), or saliva from an NPC patient (GD1). The isolate M-ABA harvested from a marmoset lymphoblastoid cell line (LCL) may deviate from the original NPC-derived virus [95]. Half (18/33) of the NPC-derived EBV genomes (denoted HN1-HN18) were contributed by one study in 2017 in which the EBV genomes have been deposited in National Center for Biotechnology Information (NCBI) but annotation for the LMP1 gene and typing for the LMP1 strain was not available [91]. Consistent with the conclusion from earlier HTA studies, China1 was indeed the predominant strain reported by HTS in NPC tumors (Table 1) [83]. 

**Figure 2:**
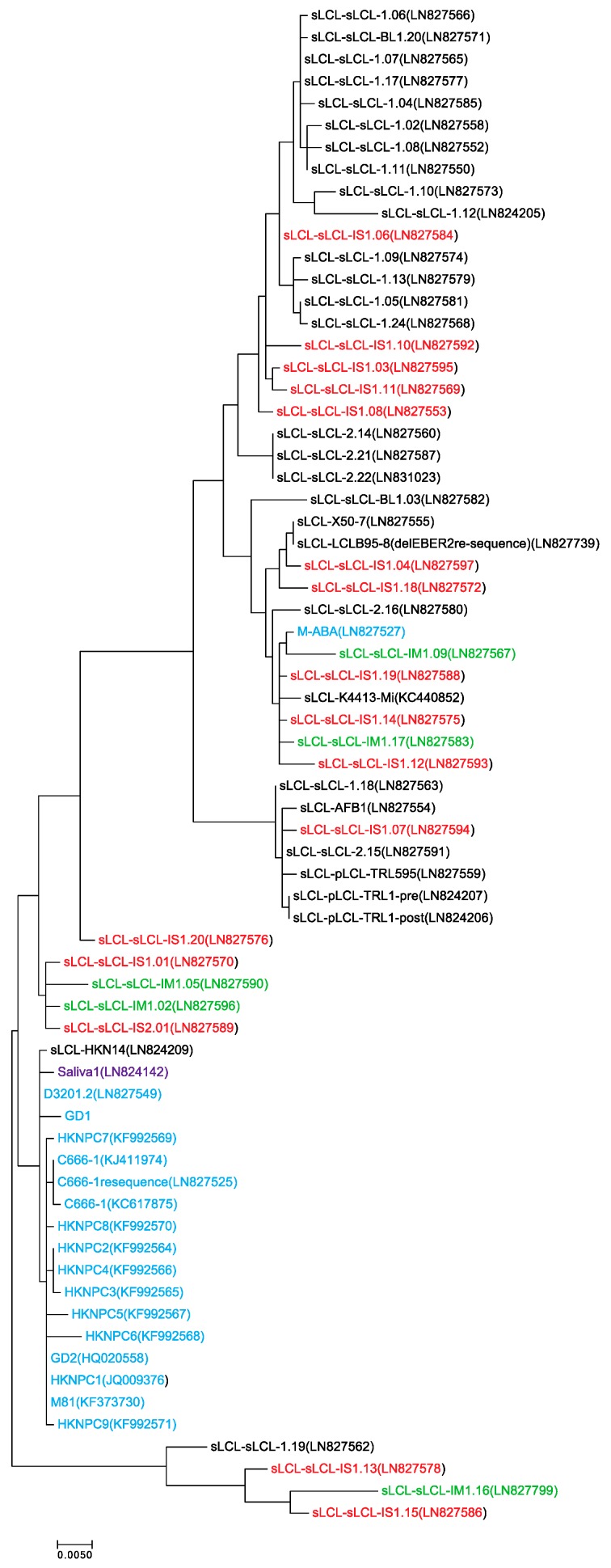
Maximum-likelihood phylogeny of LMP1 sequences. LMP1 sequences were derived from NPC patients (blue) or spontaneously immortalized lymphoblastoid cell lines (sLCLs) (black), which were further grouped by infectious mononucleosis (green) and post-transplant lymphoproliferative disease (red). One sample was directly sequenced from the saliva of a healthy donor (purple). The evolutionary history was inferred by using the Maximum Likelihood method based on the Tamura-Nei model in MEGA7 [100,101]. The tree is to scale, with branch lengths measured in the number of substitutions per site. The analysis involved 69 informative nucleotide sequences. All positions containing gaps and missing data were eliminated, leaving a total of 991 positions in the final dataset.

In samples where LMP1 annotation was available from assembled EBV genomes, a phylogenetic tree of LMP1 nucleotide sequences was constructed from NPC isolates as well as LCL isolates that represent circulating EBV variants derived from peripheral blood (Figure 2). All LCL-derived LMP1 isolates except for AFB1, which was immortalized in the presence of aflatoxin B1, were derived from spontaneously-immortalized LCLs (sLCLs) from healthy, infectious mononucleosis (IM) or post-transplant lymphoproliferative disease (PTLD) donors [90,99]. Almost all NPC-derived LMP1 sequences clustered together, except for M-ABA which is likely selected for B-cell immortalizing properties during in vitro propagation of the NPC-derived virus and was therefore not included as a representative NPC isolate in further analysis (Figure 2) [90,92,93,95,96,97,98]. 

**Figure 3:**
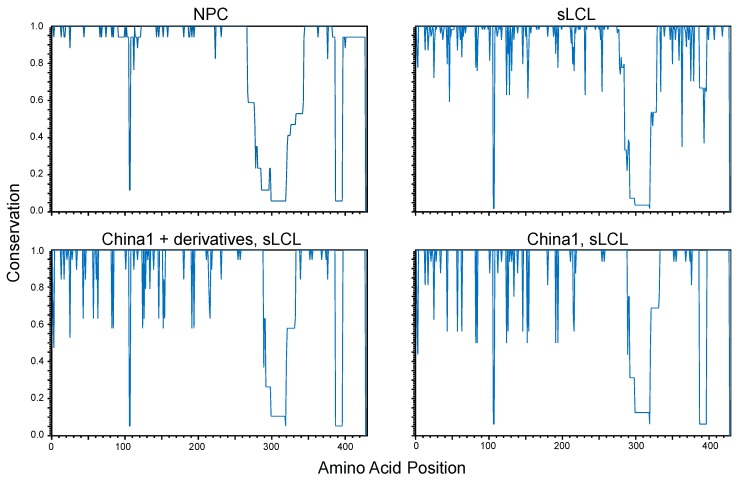
Conservation plots of LMP1 amino acid residues translated from annotated EBV genomes derived from NPC tumors (NPC), spontaneous LCLs (sLCL), and sLCLs typed with China1 LMP1 sequence (China1, sLCL) or, China1 and derivative hybrid sequences (China1 + derivatives, sLCL) [37,90,92,93,96,97,98]. Conservation plots were generated in CLC Main Workbench 6 and compared to a consensus sequence. A consensus sequence was generated from the combined sequences of NPC and sLCL samples by majority with no gaps and was included in all conservation plots to constrain the number of amino acids to the same total number in all comparisons.

In a subset of NPC samples, it was proposed that nonsynonymous mutations may cluster at the N-terminal transmembrane domains as the C-terminal domains are likely conserved for preservation of LMP1 signaling function [92]. Conservation plots of LMP1 amino acid sequences were grouped by origin (NPC vs. sLCL) and analyzed against a master consensus sequence generated from the combined NPC and sLCL sequences. Due to indels in some of the LMP1 isolates, an ungapped majority-rule consensus sequence was generated to enable comparisons among specimen groups. The conservation plots demonstrate that the coding sequence is relatively well conserved in NPC isolates but is more diverse in sLCL isolates (Figure 3). Apart for specific nucleotides in the N-terminus and one region spanning the C-terminus, the overall conservation in NPC isolates would suggest that evolutionary pressures are imposed on LMP1 in sites other than the signature amino acid loci (S229, R334, del344) that define the China1 strain [84]. All LMP1 sequences in the NPC group were typed to be the China1 strain, but the sLCL group consisted of multiple LMP1 strains [90]. It is plausible that the high overall LMP1 sequence conservation in the NPC group may simply be explained by the prevalence of the China1 strain in NPC tumors [83]. The sLCL samples were further stratified by the LMP1 strain and grouped into sLCLs encoding the China1 strain (China1, sLCL) or, sLCLs encoding China or China1-derivative hybrid LMP1 sequences (China1+derivatives, sLCL). The conservation plots of the China1 sLCL sub-groups demonstrate that LMP1 amino acid residues are more conserved in the NPC group than the sLCL isolates, suggesting that there may indeed be selection for tumor-associated residues even within the China1 clade.

Since sLCLs are cultured in vitro, it should be considered that the EBV isolates from sLCLs may not represent the LMP1 sequence diversity in circulating isolates directly harvested from biospecimens. To date, only one completed EBV genome from the saliva of a healthy asymptomatic donor has been sequenced and deposited in the National Center of Biotechnology Information (NCBI) database [90]. Targeted sequencing of the LMP1 gene to monitor intra- and inter-host variation over time in distinct anatomical compartments (oral and peripheral B-lymphocytes) from acute infectious mononucleosis and convalescence carriers, have also begun to be deposited in the NCBI database [89]. As more EBV sequences from patient samples are being deposited, a more robust analysis of the LMP1 sequence diversity found within tumors and among asymptomatic carriers will be possible.

### 4.3. Elucidation of LMP1 Selection in NPC Tumors

There are C-terminal signaling motifs that are 100% conserved in all LMP1 isolates: the PxQxT motif in CTAR1 that interacts with TRAFs, the 33bp repeat [DNGPQDPDNTD] in CTAR3 that contributes to interaction with the SUMO-conjugated enzyme Ubc9, and the YYD motif in CTAR2 that interacts with TRADD and RIP [17]. Cellular and viral determinants have been proposed as potential LMP1 selection mechanisms including immune evasion properties, oncogenic potential, and effects on EBV pathogenesis [20,26,81]. Many, but not all, of the known and predicted HLA-A2 restricted epitopes were changed in the China1 strain variant suggesting that the LMP1 sequence in NPC tumors may have been selected for immune evasion properties [81]. Many of the computer-predicted HLA-A24 restricted epitopes on LMP1 were also mutated in the China1 sequence [81]. Furthermore, although HLA-A11 is the most frequent haplotype in Chinese populations, -A2 or -A24 alleles, which are also prevalent in the general population, are the most frequently typed HLA in NPC patients [81,102,103,104]. Serological screens for LMP1 reactivity would argue that LMP1 is not an immunodominant EBV protein but is nevertheless one of the most immunogenic latent proteins expressed in the restricted latency II program in NPCs [105,106]. Clinical trials infusing autologous-CTLs targeted at LMP1 and LMP2A in NPC patients have shown promise, some with complete response [105,107]. This would support that LMP1 expression in NPC is indeed subjected to immune surveillance. A thorough analysis of HLA-type together with experiments testing HLA-presentation with donor-matched LMP1 sequences would be necessary to conclude if polymorphisms that are conserved in NPC tumors are selected for immune evasion properties.

Several studies have tested for differences in LMP1 oncogenic signaling and biological properties, including the transformation of Rat-1 fibroblasts and increased motility in human foreskin keratinocytes [20]. All LMP1 strains have oncogenic properties and while there may be notable differences in regulators of NFkB signaling, there has been no consensus on a clear difference that distinguishes the oncogenic properties of the China1 strain or the closely related CAO variant [20,108,109,110,111,112]. However, it remains possible that LMP1 sequence polymorphisms outside the regions defining LMP1 strains may indeed differ in biological properties.

A more recent appreciation of LMP1 function in epithelial infection is from elucidating its effects on the life cycle of EBV. In polarized normal oral keratinocyte cell lines, LMP1 complements cellular transcription factors to trigger the expression of lytic switch proteins Z and R [27]. Furthermore, LMP1 is required for the induction of EBV lytic genes and the efficient production of progeny virus in polarized NPC-derived epithelial cells [26]. The increased expression levels of LMP1 during EBV lytic reactivation may therefore be important in differentiation-induced EBV reactivation in oral stratified epithelium and the disease-associated permissive infection of OHL [23,26,27]. Paradoxically, LMP1 expressed in latency may also serve to suppress spontaneous reactivation through CTAR3-mediated sumoylation of KRAB-associated protein 1 (KAP1), although this may be a consequence of selective removal of LMP1 function [113]. Since LMP1 is expressed in type II latency, as well as during lytic reactivation, it is conceivable that LMP1 has roles in both latent and lytic phases of the EBV life cycle, albeit possibly different effects depending on the cellular context and the presence of other EBV transcripts.

## 5. Effect of LMP1 on Nuclear Processes and Extracellular Interactions

### 5.1. Nuclear Processes

The coding sequence of LMP1 has no overall sequence identity or homology to hosts or other viral proteins [13,17]. Furthermore, LMP1 is not known to possess intrinsic enzymatic activity [28]. Therefore, LMP1 mechanisms have been elucidated from protein-protein interactions and activation of cellular signaling pathways [6,114]. Protein-protein interaction studies originally by yeast-two-hybrid, as well as biochemical fractionation, have concluded that LMP1 signals from lipid raft microdomains and recruits TNF-receptor associated factors (TRAF 2/3/5/6) and TRADD molecules [115,116,117]. Although LMP1 localizes primarily in cytoplasmic and plasma membrane domains, one manner by which LMP1 can impact nuclear processes is by activating cellular proteins that shuttle into the nucleus. One of the most well studied LMP1-modulated nuclear processes is the activation of NFkB and STAT3 transcription factors [6,112,118,119,120,121,122,123]. Somewhat unprecedented, even integral membrane proteins induced by LMP1 and elevated in NPC, such as EGFR that is conventionally associated with the plasma membrane, can translocate into the nucleus [124,125,126]. LMP1-induced nuclear EGFR together with STAT3 binds to the Cyclin D1 promoter [127]. Coincidentally, Cyclin D1 is over-expressed in NPC tumors and promotes the stable outgrowth of EBV-infected epithelial cell clones upon de novo infection in vitro [66]. More recently, several studies have acknowledged the effect of LMP1 on nuclear processes [19,26,27,128]. LMP1 co-expressed with LMP2A modulates the DNA damage signaling protein γH2AX [19]. Furthermore, LMP1 expression impairs G2-M checkpoint in epithelial cells, which in the presence of unrepaired DNA damage could result in genomic instability [129]. LMP1 also has the potential to regulate the retention and the reactivation state of the EBV genome [26,27]. Deletion of LMP1 promotes the retention of EBV genomes in latently-infected cells, which would otherwise be evicted from cells cultured for 1–2 months in serial passage [26]. Furthermore, native expression of LMP1 contributes to EBV lytic reactivation in differentiating epithelial cells and immortalized keratinocytes [26,27].

Historically, only a handful of LMP1 interactions have been identified by yeast-2-hybrid, affinity-purification, and biomolecular fluorescence complementation (BiFC) approaches [115,130,131,132]. These conventional methods, although complementary, are reliant on stable protein-protein interactions and susceptible to stringent wash conditions. LMP1 signaling interactions are likely transient and therefore it is more challenging to identify protein networks using conventional approaches [17,116]. A proximity-based BioID (proximity-dependent biotin identification) approach in which LMP1 was fused to a promiscuous biotin ligase such that proteins in proximity (~10 nm) are labeled by biotinylation and captured for identification by mass spectrometry, has recently been applied to profile the LMP1 interactome [133]. In addition to the known interactions including LMP1-LMP1 homo-oligomers and LMP1-TRAF2 interactions, many more proteins (>1000) were identified by BioID that could traffic or potentially interact (directly or indirectly) with LMP1 [133]. The majority of identified LMP1-interacting proteins were categorized to be cytoplasmic, but nuclear proteins were also identified which could include proteins that shuttle between the cytosol and the nucleus, or are exclusive to the nucleus [133]. It is feasible that LMP1 as a transmembrane protein may shuttle into the nucleus via the nuclear envelope which is contiguous with the endoplasmic reticulum. However, it is more likely that at least some of these potential nuclear partners engage LMP1 in the cytosol and then shuttle into the nucleus. Biochemical fractionation of cellular compartments has also generated some evidence that LMP1 fractionates in the nuclear compartment, although it may be the result of contaminating endoplasmic reticulum that can co-purify with the nuclear fraction [134,135]. Complementary methods such as high resolution live cell imaging and nuclear trapping studies would be important to help resolve if a subset of LMP1 molecules may indeed shuttle into the nucleus via the nuclear envelope. The elucidation of extensive LMP1 protein interactions networks may help to unravel the mechanism(s) for how LMP1 affect nuclear processes [114,133].

### 5.2. Extracellular Interactions

#### 5.2.1. Exosomes

In addition to biological processes inside the cell, LMP1 can exert extracellular effects through delivery of LMP1-modulated extracellular vesicles (EVs) and, by modulating cell-cell and cell-extracellular matrix interactions [6,17]. Along with other EBV latent transcripts including the EBV microRNAs, LMP1 traffics with the biogenesis machinery of multivesicular bodies (MVBs) and is packaged into small EVs called exosomes [136,137,138]. LMP1-containing exosomes can be detected in the serum of NPC patients and the serum of mice transplanted with NPC xenografts [139,140]. Distinct from the localization of LMP1 to lipid rafts, cellular proteins that are co-packaged into exosomes, including the CD63 tetraspanin and ubiquitin C-terminal hydrolase L1 (UCH-L1) protein, are associated with LMP1 and required for the loading of LMP1 into exosomes [137,141,142]. The recent BioID study of LMP1 revealed that many proteins which directly or indirectly bind to LMP1, are found in exosomes or are known to play a role in exosome biogenesis and protein trafficking. Therefore, it is proposed that LMP1 hijacks protein trafficking and exosome biogenesis pathways, of which syntenin-1 and ALIX belonging to the syndecan-syntenin-ALIX axes have been verified to be important in LMP1 exosomal packaging [133]. Furthermore, crosstalk between endosomal and autophagosomal pathways can fine tune the levels of LMP1-induced intracellular signaling including activated mTOR, counterbalanced by the secretion of LMP1-modified exosomes [143]. LMP1 alters the cargo and the production of exosomes, which are thought to communicate with neighboring recipient cells in the NPC microenvironment [136,137,138,140,142,144]. LMP1 and modified exosomal content derived from both B cells and epithelial cells, have the potential to deliver phenotypic changes such as enhanced cell growth in recipient cells [145,146]. Activated signaling molecules such as the angiogenic factor FGF-2, are upregulated and released from LMP1-expressing producer cells [144]. Some of these LMP1-activated signaling proteins such as AKT, ERK and HIF-1α originating from epithelial cells can be delivered into recipient epithelial and endothelial cells, although the magnitude of biological consequence on recipient cells may depend on cumulative effects from continued exposure as would occur in vivo [140,147]. Originally described for LMP1-modified exosomes from B cells, LMP1-modified exosomes from epithelial cells can also deliver immunosuppressive cargo such as galectin 9 to disarm EBV-specific T cells that abundantly infiltrate NPC tumors [148,149,150]. In addition to the increased production of pro-inflammatory cytokines including IL6 and IL8, LMP1 also upregulates the secretion of chemokines [17,151,152,153]. NPC tumors emerge despite the pro-inflammatory tumor microenvironment with abundant T cell infiltrate, suggesting that EBV infection confers immunosuppressive effects [154,155]. The findings in exosomes would offer one mechanism by which LMP1 hijacks the EV sorting machinery to mediate intercellular communication and immune suppression in the local tumor microenvironment, and potentially exert longitudinal systemic effects. The physical properties of exosomes also differ widely from secreted cytokines and chemokines in that exosomes are stable particles (~100 nm) circulating in biofluids, carrying protein and transcripts of host and viral origin [136,137,138]. Exosomal contents are protected from enzymatic degradation and can be harvested from serum, saliva, and urine. Thus, LMP1-loaded exosomes may have the potential to be developed as non-invasive biomarkers.

#### 5.2.2. Extracellular Interactions

In addition to the indirect effects of exosomes and cytokines, LMP1 communicates directly with neighboring cells and the extracellular matrix (ECM) [17]. LMP1 alters cell-cell interactions through modifying components of adherens junctions including the upregulation of N-cadherin and downregulation of E-cadherin, known as the cadherin switch [156,157,158]. Plakoglobin, also known as γ-catenin, is a component of adherens junctions and desmosomes, whose downregulation is associated with enhanced migration [76]. LMP1 expression is sufficient to confer downregulation of junctional plakoglobin [156]. The cadherin switch is consistent between studies and cell lines, and has been documented in the majority of NPC tissues [156,158,159]. Modification of junctional proteins is important in promoting cellular migration, but the wider implications applicable to metastasis are likely to contribute to a phenomenon known as epithelial-mesenchymal transition (EMT) [160,161]. The cadherin switch and induction of vimentin and fibronectin are features of EMT and LMP1 induces all these characteristics [116,156,162,163]. Cadherin 6, also known as K-cadherin, is an EMT marker and is also induced by LMP1 expression [164]. Indeed, studies on LMP1 have shown that LMP1 can directly trigger EMT by inducing two of the four major EMT transcriptional regulators Twist and Snail [165,166].

Integrins are points of cellular adhesion to the ECM that coordinate the assembly of focal adhesions with cytoskeletal reorganization, leading to intracellular signaling and cellular migration. Fibronectin is a major constitute of the ECM and is upregulated by LMP1 leading to the ligation and assembly of integrin α5-containing focal adhesions [162,163]. Ligation of integrins activates intracellular Src and ERK signaling pathways, and are thought to contribute to LMP1-induced cellular adhesion and motility [162,163]. Intriguingly, fibronectin over-expression in NPC correlates with LMP1 expression [167]. LMP1 can also indirectly modify the extracellular matrix by increasing the production of matrix metalloproteinases (MMPs) and the large cell surface glycoprotein Mucin 1, which are thought to contribute to the degradation and dissociation from the ECM to promote metastatic invasion [168,169]. The biological consequence of all these LMP1-modulated interactions is likely to control cellular motility, invasiveness and trigger outside-in signaling. Collectively, these studies illustrate that LMP1 has multiple mechanisms to target the complex series of cell-cell and cell-ECM interactions that are dynamically regulated in metastasis.

## 6. Concluding Remarks

Arguably one of the biggest challenges in relating LMP1 research to NPC pathogenesis is the limited experimental systems that tests for LMP1 biology in epithelial infection. The recent advances in epithelial infection and polarized culture models are likely the key to fully appreciating the contribution of LMP1 to NPC pathogenesis. Intriguingly, a recent study in HPV identified striking sequence conservation in the HPV E7 oncoprotein in high-risk HPV strains [170]. Although EBV infection in NPC is distinct from the integrated HPV genome in cervical carcinoma, perhaps similar lessons could be learned from dissecting LMP1 sequence diversity. Furthermore, while it is appreciated that EBV strains with different tropism and infection efficiency may be attributed to sequence polymorphisms in multiple genes, LMP1 may be uniquely poised for functional mapping studies that have now extended beyond its recognized oncogenic properties to effects in EBV pathogenesis [171].

## Figures and Tables

**Table 1:** Epstein-Barr virus (EBV) genomes sequenced from nasopharyngeal carcinoma (NPC) patient specimens.

NPC Genome	NCBI Accession No.	Geographic Region	LMP1 Strain (If Known)	Source
GD1 [96]	AY961628	China	China 1	Saliva
GD2 [97]	HQ020558	China	China 1	NPC
HKNPC1 [92]	JQ009376	Hong Kong	China 1	NPC
C666-1 [90,98]	KC617875	China	China 1	NPC cell line
M81 [37]	KF373730	Hong Kong	China 1	NPC
D3201.2 [90]	LN827549	China	China 1	NPC
M-ABA [90]	LN827527	North Africa	B95-8	LCL, NPC virus
HN1;HN2;HN3;HN4; HN5;HN6;HN7;HN8; HN9;HN10;HN11;HN12; HN13;HN14;HN15;HN16; HN17;HN18 [91]	AB850643;AB850644;AB850648;AB850649 AB850652;AB850653;AB850655;AB850657 AB850659;AB850645;AB850646;AB850647 AB850650;AB850651;AB850654;AB850656 AB850658;AB850660	South China (Hunan Province)		NPC
HKNPC2-HKNPC9 [93]	KF992564-KF992571	Hong Kong	China 1	NPC

NCBI, National Center for Biotechnology Information; LMP1, latent membrane protein 1; LCL, lymphoblastoid cell lines.
